# Supplementary material for: Association of type 2 Diabetes Mellitus and bone mineral density: a two-sample Mendelian randomization study
Source: BMC Musculoskelet Disord. 2024 Feb 12;25:130. doi: 10.1186/s12891-024-07195-6 (PMC10860277; doi:10.1186/s12891-024-07195-6)
Supplement: Supplementary file 2 — Supplementary Material 2 [file 12891_2024_7195_MOESM2_ESM.docx]

***Supplementary Material***

**Supplementary Data**

Following Guides by STROBE-MR: Guidelines for strengthening the reporting of Mendelian randomization studies

1. TITLE and ABSTRACT

Manuscript title

Association of type 2 diabetes Diabetes Mellitus and bone mineral density: A two-sample mendelian randomization study

Abstract

Aims:

Observational studies have suggested that type 2 diabetes Diabetes Mellitus (DM2) is potentially modifiable risk factors for lower BMD, but the causal relationship is unclear. This study aimed to examine whether association of type 2 diabetes with lower BMD levels were causal by using Mendelian randomization (MR) analyses.

Methods:

We collected genome-wide association study data for DM2 and BMD of total body and different skeletal sites from the IEU database. Subsequently, we performed two-sample Mendelian randomization analysis using the TwoSampleMR package.

Results:

We identified a positive association between DM2 risk (61,714 DM2 cases and 596,424 controls) and total BMD, and other skeletal sites BMD, such as femoral neck BMD, ultradistal forearm BMD and heel BMD. However, non-significant trends were observed for the effects of DM2 on lumbar-spine BMD.

Conclusions:

In two-sample MR analyses, there was positive causal relationship between DM2 and BMD in both overall samples. In summary, although some observational analysis showed a robust association of DM2 with low BMD, there was a robust association of DM2 with high BMD as suggested by MR analysis.

2. Background

Being the prevailing metabolic ailment, DM2 has a significant threat to aging populations due to its array of complications. Apart from being a strong risk factor for cardiovascular diseases, DM2 may also increase the risk of developing osteoporosis. To date, the potential relationship between diabetes and osteoporosis has been recognized in animal^[7]^. Nonetheless, contrary to the consistently favorable outcomes observed in laboratory investigations, clinical studies have yielded incongruent findings. A meta-analysis of 15 observational studies found that individuals with DM2 from both genders have higher BMD levels^[8]^. And some cross-sectional or case-control studies also have not effectively explored the autonomous correlation between diabetes mellitus and osteoporosis^[9]^. The inconsistency could stem from significant individual variations among patients in clinical research, or it might be attributed to the absence of a clinical correlation between diabetes and BMD levels. In order to thoroughly investigate the association between the DM2 and BMD levels, a research approach more effective than RCT is required.

Mendelian randomization (MR) studies, which use an epidemiological approach that assesses the causal effect of a risk factor on an outcome, have been increasingly used to overcome the aforementioned limitations and explore causal relationships^[10]^. Since genetic variants are randomly assigned, the confounding factors are minimized by the MR method. Genetic variation significantly associated with exposure can therefore be used as instrumental variables (IVs). For instrumental variables to be valid, three conditions must be met: IV1, which is linked to the exposure; IV2, which remains unrelated to the outcome when considering the exposure; and IV3, which maintains independence from all known confounding factors up to the present. (Fig. 2) Currently, a dearth of substantial evidence exists concerning the causative factors underlying osteoporosis. Nevertheless, limited studies have concentrated on investigating the connection between DM2 status and BMD through the utilization of MR analysis.

3. Objectives

Our aim is to investigate the potentially of the relationship between DM2 and BMD levels using a large-scale genome-wide association study (GWAS) data sets by two-sample MR study. We hypothesize that DM2 is a causal risk factor for increased BMD levels. This study may help to reveal the genetic characteristics and biological mechanisms of DM2 and BMD.

METHODS

4. Study design and data sources

We first performed two-sample MR to assess the causal relationship between DM2M and BMD. A two-sample MR analysis was applied to explore the causal effects of the DM2 on BMD as our experimental flow chart shows in Fig. 1.

Note: MR, Mendelian randomization; BMD, bone mineral density; LD, linkage disequilibrium; SNPs, Single-nucleotide polymorphisms.

We acquired the summary statistics of the DM2 from a meta-analysis with ~16 million genetic variants in 62,892 DM2M cases and 596,424 controls of European ancestry^[11]^. Analysis was adjusted for age, sex, and the first 20 PCs. Genetic instruments for BMI were identified using results from the largest available meta-analysis of GWAS in 681,275 individuals of European ancestry^[11]^.

We used BMD data from a meta-analysis with five different skeletal sites as outcomes, including TB-BMD (n = 56,284), FN-BMD (n = 32,961), UF-BMD (n = 21,907), H-BMD (n = 426,824), LS-BMD (n = 28,498)^[12]^. TB-BMD, FN-BMD, FABMD and LS-BMD were measured by DXA, whereas H-BMD was measured by quantitative ultrasound (QUS), which are two completely different procedures.

5. Assumptions

A two-sample MR method should conform to three fundamental assumptions: filtered IVs must be strongly related to the DM2; filtered IVs are not correlated with confounding factors; IVs can only influence BMD through the DM2. (Fig. 2) We used publicly available GWAS data with the informed consent and ethical approval previously obtained.

6. Statistical methods: main analysis and MR estimation

For two-sample MR analysis, a genome-wide single-nucleotide polymorphisms (SNPs) significantly associated with DM2 (p < 5 × 10^−8^). Then, we pooled all genome-wide significant SNPs that were significantly associated with DM2 and then clumped these SNPs with respect to the lowest p-value corresponding to any of the two using a 1,0000-kb window and pairwise LD r ^2^ < 0.01. We calculated the proportion of phenotypic variance explained by instrumental variable SNPs of DM2 and computed the F-statistic (>10) to verify whether they were strong instruments.

We used the inverse-variance weighted (IVW) method as the primary MR approach^[13]^. MR-Egger, weighted median, and weighted mode further conducted to MR analysis. However, the estimation accuracy produced by MR-Egger is very low. Weighted median gives an accurate estimate based on the assumption that at least 50% of IVs are valid^[14]^. Weighted mode is sensitive to the difficult bandwidth selection for mode estimation^[15]^.

7. Assessment of assumptions

Based on the MR models and pleiotropy assessments mentions above, we considered a relatively robust inference meeting the following items: (i) MR analyses presented a consistent direction of casual estimates among different methods. (ii) Intercept term derived from MR-Egger regression suggested no directional pleiotropy detected (p > 0.05). (iii) Leave-one-out analysis suggested causal estimate was not strongly driven by a single SNP.

8. Sensitivity analyses

Sensitivity analysis has been pivotal in MR studies to detect underlying heterogeneity and pleiotropy for MR estimates can be severely violated. We used the Cochran Q statistic and leave-one-out analysis to test for the heterogeneity, and Egger-intercept to test for the pleiotropy^[13, 16]^. And if there is heterogeneity, random-effects IVW models are applied; otherwise, the fixed-effect IVW model is applied^[17]^.

9. Software and pre-registration

All statistical analyses were conducted using the “Two Sample MR” (version 0.5.7, Stephen Burgess, Chicago, IL, USA) and “Mendelian Randomization” (version 0.8.0) in the statistical program R (version 4.3.1). p < 0.05 was considered as statistically significant. The study protocol and details were not pre-registered.

RESULTS

10. Descriptive data

11. Main results

Causal Effect of Increased DM2 on TB-BMD

The specific MR data for all selected SNPs in the exposure set (DM2) and outcome set (TB-BMD) are presented in Supplementary Table S1. IVW analysis showed that there was a positive causal association between DM2 and TB-BMD (p = 0.002, OR = 1.03) (Table 1). MR Egger analysis showed that there was not a causal association between DM2 and TB-BMD (p = 0.33, OR = 1.02) (Table 1). Weighted median analysis demonstrated that DM2 had a positive causal association with TB-BMD (p < 0.05, OR > 1) (Table 1). Weighted mode analysis demonstrated that DM2 had a positive causal association with TB-BMD (p < 0.05, OR > 1) (Table 1). The estimated effect sizes of the SNPs on both the DM2 (exposure) and TB-BMD (outcome) are presented in scatter plots (Figure S1A). Funnel plot presents symmetrical distribution. (Figure S1B) Plots of the leave-one-out analysis, as shown in Figure S1C, demonstrates that no potentially influential SNP that drive the causal effect.

Causal Effect of Increased DM2 on FN-BMD

The specific MR data for all selected SNPs in the exposure set (DM2) and outcome set (FN-BMD) are presented in Supplementary Table S2. IVW analysis showed that there was a positive causal association between DM2 and FN-BMD (p = 0.001, OR = 1.04) (Table 1). MR Egger analysis showed that there was not a causal association between DM2 and FN-BMD (p = 0.12, OR = 1.05) (Table 1). Weighted median analysis demonstrated that DM2 had no causal association with FN-BMD (p < 0.05, OR > 1) (Table 1). weighted mode analysis demonstrated that DM2 had a positive causal association with FN-BMD (p < 0.05, OR > 1) (Table 1). The estimated effect sizes of the SNPs on both the DM2 (exposure) and TB-BMD (outcome) are presented in scatter plots (Figure S2A). Funnel plot presents symmetrical distribution. (Figure S2B) Plots of the leave-one-out analysis, as shown in Figure S2C, demonstrates that no potentially influential SNP that drive the causal effect.

Causal Effect of Increased DM2 on UF -BMD

The specific MR data for all selected SNPs in the exposure set (DM2) and outcome set (UF-BMD) are presented in Supplementary Table S3. IVW analysis showed that there was a positive causal association between DM2 and UF-BMD (p = 7e^-4^, OR = 1.05) (Table 1). MR Egger, weighted median, and weighted mode analysis showed that there was not a causal association between DM2 and UF-BMD (p > 0.05, OR > 1) (Table 1). The estimated effect sizes of the SNPs on both the DM2 (exposure) and UF-BMD (outcome) are presented in scatter plots (Figure S3A). Funnel plot presents symmetrical distribution. (Figure S3B) Plots of the leave-one-out analysis, as shown in Figure S3C, demonstrates that no potentially influential SNP that drive the causal effect.

Causal Effect of Increased DM2 on H -BMD

The specific MR data for all selected SNPs in the exposure set (DM2) and outcome set (H-BMD) are presented in Supplementary Table S4. IVW analysis showed that there was a positive causal association between DM2 and H-BMD (p = 4.3e^-4^, OR = 1.03) (Table 1). MR Egger analysis showed that there was not a causal association between DM2 and H-BMD (p > 0.05) (Table 1). Weighted median analysis and weighted mode analysis demonstrated that DM2 had a positive causal association with H-BMD (p < 0.05, OR > 1) (Table 1). The estimated effect sizes of the SNPs on both the DM2 (exposure) and H-BMD (outcome) are presented in scatter plots (Figure S4A). Funnel plot presents symmetrical distribution. (Figure S4B) Plots of the leave-one-out analysis, as shown in Figure S4C, demonstrates that no potentially influential SNP that drive the causal effect.

Causal Effect of Increased DM2 on LS -BMD

The specific MR data for all selected SNPs in the exposure set (DM2) and outcome set (LS-BMD) are presented in Supplementary Table S5. IVW analysis showed that there was a positive causal association between DM2 and LS-BMD (p = 0.003, OR = 1.05) (Table 1). Weighted median analysis showed that there was not a causal association between DM2 and LS-BMD (p > 0.05, OR > 1) (Table 1). MR Egger and weighted mode showed that there was not a causal association between DM2 and LS-BMD (p > 0.05, OR < 1) (Table 1). The estimated effect sizes of the SNPs on both the DM2 (exposure) and LS-BMD (outcome) are presented in scatter plots (Figure S5A). Funnel plot presents symmetrical distribution. (Figure S5B) Plots of the leave-one-out analysis, as shown in Figure S5C, demonstrates that no potentially influential SNP that drive the causal effect.

13. Sensitivity and additional analyses

Sensitivity analysis was conducted to verify the reliability of IVW results. IVW and MR-Egger test for heterogeneity showed that there was no heterogeneity in MR analysis results between DM2 and TB-BMD, FN-BMD, UF-BMD, H-BMD and LS-BMD (p > 0.05) (Table 2). And the funnel plots present symmetrical distribution. (Figure S1C-S5C)

Further Validation of MR Results

We further verified the IVW results. The results of IVW (fixed effects), weight median and weight median mode revealed that DM2 had positive causal association with TB-BMD (p < 0.05, OR > 1) and H-BMD (p < 0.05, OR > 1) (Figure 3). The results of IVW (fixed effects) and weight mode demonstrated a positive causal association between DM2 and FN-BMD (p < 0.05, OR > 1) (Figure 3). However, the other MR methods cannot verify the IVW results about DM2 and UF-BMD and DM2 and LS-BMD (p > 0.05) (Figure 3), indicating that the results about DM2 having a positive causal connection with UF-BMD and LS-BMD are unstable.

DISCUSSION

14. Key results

Type 2 diabetes (DM2) influences bone metabolism, but the relation of DM2 with bone mineral density (BMD) remains inconsistent across studies. Observational studies provide evidence for higher fracture risk for a given BMD in individuals with DM2 compared to those without this condition^[18]^. Despite this higher fracture risk, some observational studies found that individuals with DM2 tend to have a higher BMD than non-diabetic individuals^[19, 20]^. In a meta-analysis of 3,437 subjects with DM2 generally have higher BMD as compared with healthy controls, with significant differences of 0.04 (95%CI: 0.02, 0.05) at the femoral neck, 0.06 (95%CI: 0.04, 0.08) at the hip and 0.06 (95%CI: 0.04, 0.07) at the spine^[8]^. What is the association between DM2 and BMD? Because of confounding in observational studies between DM2 and other metabolic factors known to influence bone homeostasis^[21]^, the precise effects of DM2 and BMD remain unclear. MR has great potential for analyzing the causal associations between diseases and traits. As far as we know, this study is the first to investigate the genetic causal associations between DM2 and different skeletal sites BMD, which provides insights into the inconsistently prior reported relationship. Our MR analysis concluded that overall individuals with DM2 have higher TB-BMD, FN-BMD, UF-BMD, and H-BMD. And the association between DM2 and LS-BMD did not reach statistical significance, raising the possibility that the effect of DM2 on BMD is site-specific. Sensitivity analyses did not essentially change our results or conclusions.

15. Limitations

There are several limitations to this research. Among the MR statistical methods, although the causal effect of exposure on outcome was consistent in both IVW and WM (weight median or weight mode) test methods, the results of MR-Egger were less convincing. And this study is confined to the European population; whether this association exists in other groups warrants additional investigation. Moreover, the precise rationale for potential measurement discrepancies having a greater impact on lumbar spine data in comparison to data from other anatomical sites remains uncertain. Additional investigations into the mechanisms that could potentially elucidate the site-specific effects of DM2 on BMD and other bone characteristics are imperative to shed light on these uncertainties.

16. Interpretation

An array of mechanisms potentially underlies the connection between DM2M and rising BMD, though their exact nature remains largely obscure. In the ensuing discussion, we approach the subject from a clinical standpoint and highlight the pivotal factors that exert substantial influence on the intricate interrelationship between DM2 and BMD.

Obesity and hyperinsulinemia have been theorized to constitute two significant attributes of DM2 that exhibit a positive correlation with BMD^[22]^. However, it was observed that a substantial proportion of the studies included did not fundamentally alter the association even after accounting for BMI. Numerous intricate pathways exist through which obesity might affect the connection between diabetes and BMD. The influence of body fatness on the precision of DXA-based BMD assessments, as evidenced in obese individuals with diabetes, is noteworthy^[23]^. However, the potential measurement error should be of minimal concern, given that this phenomenon has been demonstrated to potentially result in either underestimation or overestimation of values. Moreover, it has been shown to exert a negligible impact on the precision of BMD measurements. Conversely, adipose tissue releases an extensive array of adipokines that have been suggested to play a role, whether directly or indirectly, in the modulation of bone remodeling processes^[24]^. Plasma leptin concentrations have been shown to be higher in diabetic men than in healthy controls. Elevated plasma leptin levels have been demonstrated in diabetic men compared to their healthy counterparts^[25]^. Furthermore, it has been revealed that leptin can hinder osteoclast formation by diminishing the production of RANK/RANKL while enhancing osteoprotegerin levels^[26-29]^. And other adipokines, such as adiponectin and resistin, are also found to be present in osteoblasts and osteoclasts^[30]^ (Figure 4). The impact of these adipokines on bone metabolism remains predominantly unclear, although their potential involvement in directing the differentiation of mesenchymal progenitor cells towards either osteogenic or adipogenic pathways is noteworthy^[31, 32]^.

Several of the reviewed studies suggested that insulin levels might partially mediate the positive association between DM2 and increased BMD. Individuals with DM2 typically exhibit an excess of insulin. From a physiological perspective, insulin exerts an anabolic influence on bone, primarily attributed to its structural resemblance to Insulin-like growth factor 1 (IGF-1). This resemblance allows insulin to engage with the IGF-1 receptor, which is found on osteoblasts^[36]^. The signaling pathway of IGF-1 holds paramount importance in bone acquisition^[37]^, as evidenced by both human and mouse investigations revealing a noteworthy and affirmative correlation between IGF-1 levels and BMD^[38, 39]^. Considering this standpoint, a hypothesis arises that hyperinsulinemia might potentially trigger a mitogenic impact on osteoblasts, promoting their differentiation through the activation of the IGF-1 signaling pathway (Figure 4). Furthermore, certain indirect effects of insulin on bone formation may conceivably be mediated by osteogenic factors such as amylin, osteoprotegerin, sex steroids, and sex hormone-binding globulin.

Insulin, as an anabolic hormone, holds a pivotal role in governing substrate metabolism across critical organs and tissues, including skeletal muscle, the liver, and adipose tissue^[40]^. The insulin receptor is expressed in both osteoblasts and osteoclasts. Insulin not only triggers the formation of osteoclasts but also fosters the proliferation, differentiation, and survival of osteoblasts. This collective effect tends to tip the scale in favor of bone formation^[41]^. Research involving insulin receptor knockout mice suggests that insulin signaling is essential for optimal bone acquisition, possibly owing to insulin's involvement in governing bone energy metabolism^[42, 43]^. In fact, the administration of insulin leads to an augmentation in 18F-fluorodeoxyglucose uptake within bone tissues in mice, a response noticeably diminished in mice wherein the insulin receptor is absent in osteoblasts^[44]^. Moreover, the activation of the insulin receptor within the growth plate of mice subjected to a hypercaloric diet stimulates skeletal growth and augments the process of growth plate chondrogenesis^[45]^. Osteoblasts also express the IGF-1 receptor^[46]^. IGF-1 binds to both the IGF-1 receptor and, with a slightly lower affinity, to the insulin receptor, thereby activating the insulin signaling pathway and eliciting osteoanabolic effects. Additionally, the utilization of Thiazide, which is anticipated to be more prevalent among individuals with diabetes, has been correlated with elevated BMD at various skeletal sites^[47, 48]^. Similarly, the usage of statins (likewise more common in diabetics) has also demonstrated an association with higher BMD^[49, 50]^.

It is worth mentioning that DM2 affects BMD differently in different parts of the body. We speculated a possible mechanism for site-specific effects of DM2 on BMD could relate to the known disparate effects of DM2 on cortical and trabecular bone^[51-53]^ and the significant regional variation in bone microstructure throughout the skeleton^[54, 55]^. Alternatively, measurement error for LS-BMD due to non-osteoporotic degenerative changes in the spine (such as osteophytes and degenerative disc disease)^[56]^ or technical issues (such as positioning)^[57]^ may have biased associations towards the null. Nonetheless, the precise rationale for potential measurement discrepancies having a greater impact on lumbar spine data in comparison to data from other anatomical sites remains uncertain. Additional investigations into the mechanisms that could potentially elucidate the site-specific effects of DM2 on BMD and other bone characteristics are imperative to shed light on these uncertainties.

OTHER INFORMATION

17. Funding

Not available.

18. Data and data sharing

The article/Supplementary Material contains the original contributions presented in the study; further questions should be directed to the corresponding author(s).

19. Conflicts of Interest

Declaration of interest: none.
